# Resistant *Salmonella* Virchow in Quail Products

**DOI:** 10.3201/eid1112.010977

**Published:** 2005-12

**Authors:** Frank M. Aarestrup, Henrik Hasman, Lars Bogø Jensen

**Affiliations:** * Danish Institute for Food and Veterinary Research, Copenhagen V, Denmark

**Keywords:** Salmonella, ESBL, quinolone, letter

**To the Editor:** *Salmonella* spp. resistant to multiple antimicrobial agents have emerged worldwide in recent years, but clinical relevance varies with the agent to which resistance evolves. Fluoroquinolones are often the drug of choice to treat gastrointestinal infections in humans, and resistance to this class of antimicrobial agents is associated with increased illness and death (*1*). Resistance to fluoroquinolones has emerged worldwide during the last decade. *Salmonella* isolates resistant to oxyiminocephalosporins because they produce extended-spectrum β-lactamases (ESBLs) have emerged worldwide since 1992. This emergence has caused concern since cephalosporins are drugs of choice to treat salmonellosis in children, to whom fluoroquinolones must not be administered because of toxicity issues. In Denmark, the first ESBL-producing isolate of animal origin from a *Salmonella enterica* serovar Heidelberg isolated from a boar imported from Canada in 2003 was reported (*2*), but such isolates have not previously been reported in food products.

On October 15, 2003, the Danish Institute for Veterinary Research, the national reference laboratory, received 3 *Salmonella* isolates found in quails imported from France. *Salmonella* isolates found at any importer's laboratory in Denmark are submitted to the reference laboratory for further analyses. The quails were in the importer's storage room at the time of sampling; sampling was performed routinely by the importer's own laboratory. At the reference laboratory, the isolates were serotyped as *S*. *enterica* serovar Virchow and found resistant to ampicillin, ceftiofur, cephalothin, nalidixic acid, and tetracycline and with reduced susceptibility to ciprofloxacin (MICs >0.125 μg/mL) (*3*). Polymerase chain reaction detection and sequencing (*4*) showed that the β-lactam resistance was mediated by *bla*_CTX-M-9_. Pulsed-field gel electrophoresis was performed by using *Xba*I and *Bln*I as restriction enzymes according to the PulseNet protocol (*5*), and all 3 isolates had the same profile.

On October 23, the importer was informed of the laboratory's findings and the increased risk associated with salmonella isolates simultaneously resistant to quinolones and cephalosporins. Based on this information, the importer withdrew the product from the supermarkets on October 24. Recently, *S*. *enterica* Virchow with *bla*_CTX-M-9_ has also been reported in poultry, poultry products, and humans in France (*6*), as well as humans in Spain (*7*) and the United Kingdom (*8*). The isolates from France were also resistant to nalidixic acid; the isolates we have obtained from fresh quails imported from France are possibly related to these isolates.

The global food-products trade is expected to increase in the future. Thus, attempts to improve food safety must emphasize detection of antimicrobial drug–resistant bacteria in imported food products. Furthermore, international agreements that limit contamination with drug-resistant bacteria and resistance genes at the primary production site are necessary to ensure consumer safety (*9*). International agreements must be based on antimicrobial-resistance data and early reports of emerging problems. Recently, the World Health Organization (WHO) launched the Global Salm Surv program (*10*) to isolate and identify antimicrobial resistance to *Salmonella* globally.

Many national and international rules, as well as marketing and consumer factors, regulate the international trade of food products and live animals. Large international corporations may also affect international trade. For example, McDonald's Corporation has issued a global policy for antimicrobial drug use in food animals that specifies requirements for their food product suppliers. Local groceries or supermarkets may also impose their own standards nationally. We are aware of only 1 product withdrawal related to antimicrobial resistance, the quail imported from France.

No international standards exist for managing food safety problems related to antimicrobial resistance. However, in 2003 the Food and Agriculture Organization of the United Nations, WHO, and the World Organisation for Animal Health jointly hosted a workshop with a panel of experts to scientifically assess resistance risks related to nonhuman use of antimicrobial drugs (*9*). The panel's purpose was also to provide recommendations to the Codex Alimentarius Commission for future risk management of antimicrobial drug resistance (*9*). Imposing restrictions on products with combinations of resistance, such as simultaneous resistance to quinolones and cephalosporins in *Salmonella*, as reported in this study, would be a good first step towards managing antimicrobial drug–resistance risks.
